# iPS-Cell Technology and the Problem of Genetic Instability—Can It Ever Be Safe for Clinical Use?

**DOI:** 10.3390/jcm8030288

**Published:** 2019-02-28

**Authors:** Stephen W. Attwood, Michael J. Edel

**Affiliations:** 1 Department of Life Sciences, The Natural History Museum, London SW7 5BD, UK; 2 Department of Physiology, Anatomy and Genetics, University of Oxford, Oxford OX1 3PT, UK; edel.michael@gmail.com; 3 Control of Pluripotency Laboratory, Department of Physiological Sciences I, Faculty of Medicine, University of Barcelona, Hospital Clinic, Casanova 143, 08036 Barcelona, Spain; 4 Victor Chang Cardiac Research Institute, Sydney, NSW 2145, Australia; 5 Harry Perkins Research Institute, Fiona Stanley Hospital, University of Western Australia, PO Box 404, Bull Creek, Western Australia 6149, Australia

**Keywords:** adverse event, clinical translation, evolution, genetic stability, pluripotent stem-cell, safety, stem cell, stem-cell research, stem cell therapy

## Abstract

The use of induced Pluripotent Stem Cells (iPSC) as a source of autologous tissues shows great promise in regenerative medicine. Nevertheless, several major challenges remain to be addressed before iPSC-derived cells can be used in therapy, and experience of their clinical use is extremely limited. In this review, the factors affecting the safe translation of iPSC to the clinic are considered, together with an account of efforts being made to overcome these issues. The review draws upon experiences with pluripotent stem-cell therapeutics, including clinical trials involving human embryonic stem cells and the widely transplanted mesenchymal stem cells. The discussion covers concerns relating to: (i) the reprogramming process; (ii) the detection and removal of incompletely differentiated and pluripotent cells from the resulting medicinal products; and (iii) genomic and epigenetic changes, and the evolutionary and selective processes occurring during culture expansion, associated with production of iPSC-therapeutics. In addition, (iv) methods for the practical culture-at-scale and standardization required for routine clinical use are considered. Finally, (v) the potential of iPSC in the treatment of human disease is evaluated in the light of what is known about the reprogramming process, the behavior of cells in culture, and the performance of iPSC in pre-clinical studies.

## 1. Introduction

### 1.1. Experiences with Human Embryonic Stem-Cell (hESC)-Derived Cells

Much can be learnt from recent clinical trials involving hESC-derived cells in the treatment of degenerative diseases, such as spinal cord injury and the wet form of macular degeneration [1]. The trial data show a general lack of Serious Adverse Events (SAE) associated with cell implants derived from hESC [2], and studies of hESC lines have shed light on genomic aberrations and epigenetic changes associated with long-term in vivo culture and directed differentiation of pluripotent cells [3]. In addition, experiences with hESC have informed the development of safer and better standardized culture protocols that enhance genome stability and the fidelity of derived cells [4]. Together with Mesenchymal Stem Cells (MSC), also reviewed here, hESC provide evidence of safety across a much greater range of tissue types, transplant sites, and over longer periods of clinical follow-up than the five years possible with induced Pluripotent Stem Cells (iPSC). Understanding gleaned from review of hESC and MSC clinical deployment is, however, limited in that it reveals little about the effects of the reprogramming step, which is unique to iPSC.

Although hESC have the potential to differentiate into any adult cell type required for the therapeutic regeneration of diseased tissues, numerous concerns hinder their clinical scale-up and widespread use:

- Ethical issues surround the source of hESC, which is usually the destruction of a 5–10-day-old blastocyst―a cluster of 100–200 cells [5].
- Therapeutic use of hESCs is inherently problematic. The cells are not only potentially immunogenic, but also the use of failed In Vitro Fertilization (IVF) embryos invites complications of abnormal development.
- hESC lines often show or develop karyotypic abnormalities associated with proliferative advantage or exhibit full teratomagenicity [6].

### 1.2. Human-Induced Pluripotent Stem Cells (hiPSC)

**Figure 1:**
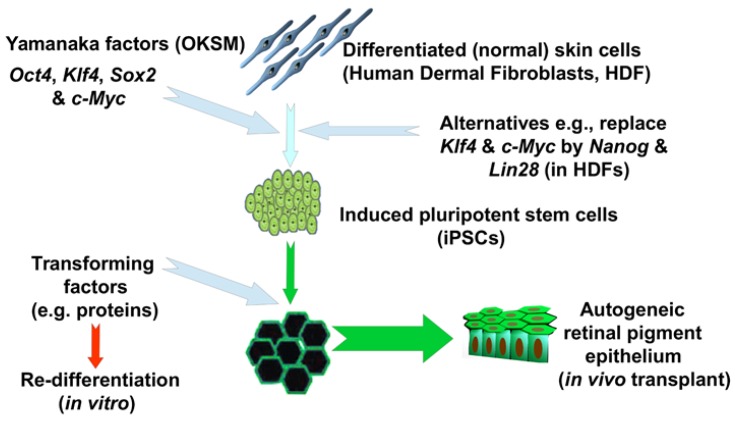
Outline of the procedure underlying the production of iPSC. The example given is of their use in treating age-related macular degeneration [11].

The discovery in 2006 that retrovirus-mediated transfection of four Embryonic Stem Cell (ESC) phenotype-related factors (subsequently termed “Yamanaka-factors” or “OKSM” (OCT4, KLF4, SOX2 and MYC) including variants thereof [7]) (Figure 1) could produce ESC-like stem cells from, first Mouse Embryonic-Fibroblasts (MEF), mouse adult [8] and then Human Dermal-Fibroblasts (HDF) [9,10], was a major step in addressing the failings of hESC-therapies. These authors called the transformed cells “induced pluripotent stem cells” as they exhibited characteristics of pluripotency.

**Figure 2:**
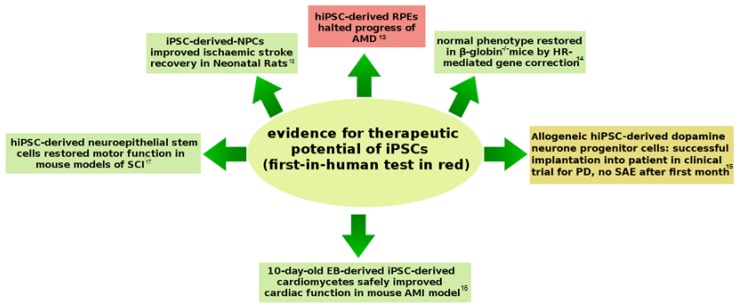
Evidence of the therapeutic potential of iPSC-derived stem cells in the form of two clinical and four pre-clinical studies. Abbreviations: AMD, Age-related Macular Degeneration; AMI, Acute Myocardial Infarction; EB, Embryoid Body; HR, Homologous Recombination; NPC, Neural Progenitor Cells; PD, Parkinson’s Disease; SAE, Serious Adverse Event; SCI, Spinal Cord Injury. Sources: [13,14,15,16,17,18].

Autologous iPSC appeared to offer new therapies for patients with a rare tissue type, or for those in cell therapies where repeated collection of stem cells is necessary (e.g., autologous blood stem-cell transplantation in myeloma chemotherapy [12]). The therapeutic potential of iPSC was demonstrated unequivocally in animals, leading up to the First-In-Human (FIH) test case in 2014 to treat Age-related Macular Degeneration (AMD) (Figure 2). In view of the current expansion of clinical trials involving iPSC, this review will consider the safety issues surrounding the use of iPSC-therapies, and how these might be overcome in the treatment of degenerative disease.

## 2. Reprogramming Leads to Genetic Dysregulation

The first hiPSC experiments used lentivirus to deliver Yamanaka-factors in transfection of HDF [9]. Such retroviral-vectors become integrated into the genome of re-implanted cells and therefore pose some risk of Insertional Mutagenesis (IM) [19]. The risk of IM was soon overcome by the development of non-integrating vectors. For example, non-integrating viral vectors were constructed, the most promising of which is the Sendai virus (SeV), a negative-strand RNA virus [20,21,22]. Several non-viral approaches were also developed that may address the main shortcomings of SeV reprogramming, such as slow clearance of SeV RNA, lack of a cGMP-grade kit (Good Manufacturing Practice certified, cGMP), and difficulty in replacing reprogramming factors (e.g., for testing alternative combinations) [23]. For example, double-stranded micro-RNAs (miRNAs) have been used to reprogram mouse Adipose Stromal Cells (ASC) and HDF (Table 1, row 1), possibly through their role in inhibiting DNA methylation, promoting mesenchymal to epithelial transition, and targeting cell-cycle regulators [24]. The approach has been hailed as least tumorigenic among the current reprogramming methods and >10% efficient [25]; however, if success in reprogramming ESC and carcinoma cells is excluded from consideration, more modest efficiencies of 0.002% are seen [26].

More promising as a safer transgene-free approach for clinical applications is direct (e.g., endocytosis within a cationic carrier) transfection with mRNAs coding for Yamanaka-factors to form RiPSC (mRNA-induced pluripotent stem cells) [27]. Generation of RiPSC is hampered by the immunogenicity of exogenous mRNAs, the high levels of cytotoxicity observed in mRNA transfections, and by the rapid degradation of exogenous mRNA (necessitating daily transfections for >2 weeks) [29]―nevertheless, solutions are emerging which range from simple 5’-guanine capping to increase half-life [27], to introduction of all reprogramming factors via a single positive-strand RNA-replicon (based on Venezuelan Equine Encephalitis (VEE)). The VEE vector needs only a single transfection; however, it is immunogenic and requires culture in the presence of interferon inhibitors [32]. Although most efficient, RiPSC production is technically challenging and works with only the least refractory of fibroblasts [29]. A Whole Genome Sequencing (WGS) study examining variation induced in nine isogenic iPSC-lines, reprogrammed using integrating retroviral-vectors, SeV, and mRNA-vectors, found only low numbers of Single-Nucleotide Variants (SNV) (between 350 and 810) relative to the founding fibroblasts, and no significant difference in number among the three vector classes [33]. Although the RiPSC showed fewer mutations than SeV or retroviral vectored iPSC, they possessed the greatest number of mutations in EZH2 binding sites, and were the only cell type to show large structural variation (a 228.8-kbp deletion), with SeV-reprogrammed cells having fewest coding mutations. RiPSC led to a similar number of mutations as retroviral-vectoring and SeV-vectoring; however, there was a greater probability of deleterious mutation with retroviral-vectoring [33]. In contrast, a similar Whole Exome Sequencing (WES) study on 22 hiPSC-lines, estimated up to three protein-coding point mutations per exome and found enrichment for mutations associated with cancer [34].

An alternative solution to the requirement for a non-integrating vector is the use of piggyBac transposons carrying the reprogramming factor cassette. PiggyBac transposons are known for their almost footprint-free excision from the genome following re-expression of a transposase [35]. The approach has been used to generate iPSC from murine fibroblasts [36] and MEF [37]. Reprogramming efficiencies as high as 1% were reported, although at high dose transfections (~0.1% at moderate doses) [37]. Clearly, the use of such transposons is promising; however, clean excision of piggyBac transposons generally occurs at a frequency of 95% [38], and 91% was reported during iPSC reprogramming [36]. In an implant of 10^9^ iPSC-derivate cells, 50 million might be expected to retain the transposon, and, as these vectors lack the natural gene-silencing character [39] of most viral vectors, this implies a potential safety issue (although an inducible promoter can be introduced, mutations therein or lack of specificity may pose a long-term risk). Further study of the stability and transcriptome of piggyBac transposon vectored iPSC-derivates is therefore necessary to determine their clinical promise in relation to other forms of non-integrating vector.

Reprogramming by mini-circle vector (mc-hiPSC), originally derived from human ASC, has been regarded as a safe form of transgene-free reprogramming; however, mc-hiPSC transcriptomics has revealed many cancer-specific features, numbering only slightly less than that seen with lentiviral-vectors [40]. Episomal plasmids already provide transgene-free iPSC, require only single transfections, and oriP/EBNA1 achieves non-integrative attachment to the host chromatin such that the plasmid replicates once with each host mitosis [41]. Consequently, episomal plasmids may be the current best option for clinical translation and were used in the FIH hiPSC trial [11]; however, they require bespoke culture conditions (e.g., E8 media, hypoxic conditions, and many factors additional to OKSM) [29]. Furthermore, treatment of the second patient in the Riken Institute’s hiPSC-derived Retinal Pigment Epithelium (RPE) trial had to be canceled following detection of three SNV and three Copy Number Variations (CNV) in the hiPSC that were not present in the patient’s own (donor) cells [42].

In addition to mRNAs, RNA viruses, and plasmids, transgene-free reprogramming has also been achieved with recombinant proteins (specifically proteins bulk produced by recombinant bacteria, such as *E. coli*, and not recombinant proteins *sensu stricto*). The approach was first demonstrated using OKSM proteins to reprogram MEF back into a pluripotent state [43] (and one month later by a second group using human fibroblasts [44]). The resulting iPSC (termed piPSC) closely resembled murine ESC in terms of proteome and DNA methylation patterns; the piPSC also formed embryoid bodies containing cells in all three germ layers, and integrated into mouse tissues in vivo. The technique was subsequently refined and developed by several research groups (e.g., [45,46,47,48,49]). Although avoiding the problem of IM, the efficiency of piPSC protocols is generally low (0.001–0.006%) [50], this being most likely due to failure of the proteins to penetrate the hydrophobic cell membrane [51]. The low cell permeability has been partly overcome through fusion of the proteins with cell penetrating peptides [44] or, more recently, by treatment of the donor cells with titanium oxide nanotubes [52], and use of bolaamphiphiles to stabilize cell membrane proteins and so facilitate uptake of the proteins [53]. In addition, reduction of cytosolic localization in endosomes, using nuclear localization signal/sequence-fused proteins, has been attempted [48]. Nevertheless, reprogramming efficiency did not rise significantly above 0.05%. Furthermore, skipping the transcription and translation stages seen in traditional iPSC production does not necessarily mean that piPSC are safer, as only transient expression of reprogramming factors has been shown to be sufficient to trigger oncogenesis in iPSC ―reprogramming using exogenous proteins is equivalent to transient overexpression of nucleic acid-based factors. Consequently, while piPSC already offer efficiencies approaching those of SeV, claims as to their much greater safety require both theoretical and empirical assessment.

**Figure 3:**
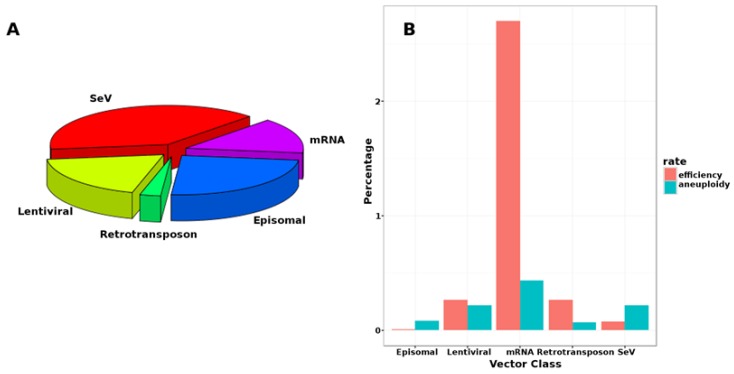
Adoption and performance of alternative vector classes. (**A**) Usage of different classes by labs generating iPSC from fibroblasts or red blood cells; (**B**) Reprogramming efficiency and reciprocal of aneuploidy rates (as percentages) for each vector. Plotted using R computing language [54] and based on data in Schlaeger et al. (2014) [23].

Despite these developments, viral vectors remain most popular among researchers (Figure 3A), probably owing to their persistence (requiring only one transfection) and ease of use (laboratories may easily share vector constructs for consistency), rather than their relative efficiency (Figure 3B).

The lengthy reprogramming, population expansion, and derivation steps of iPSC-therapeutic manufacture make autologous iPSC-derivates impractical for intervention in cases of acute disease. Consequently, “Universal” iPSC (UiPSC) were recently developed to address the problem of immunogenicity of allogeneic iPSC-derivate implants, and their production has involved a combination of several transductional and gene-editing strategies―suggesting possibly more complex safety issues. UiPSC achieve hypoimmunogenicity by mimicking the low major histocompatibility complex class I and II expression and strong expression of CD47 shown by fetal cells. Human UiPSC production currently involves subjecting an iPSC, resulting from episomal reprogramming of CD34+ cord-blood cells, to two CRISPR-Cas9 gene-editing steps, followed by lentiviral vectoring of *CD47*. Hypoimmunogenic UiPSC have also been successfully produced from murine iPSC [55]. Subjection of cells destined for downstream population expansion to double CRISPR-Cas9 editing is of concern, because even a single edit has been shown to effect selection for cells (including iPSC) lacking a functional *p53*-pathway (as a *p53*-mediated apoptotic response triggered by the double-strand DNA-break removes *p53*+ cells) [56,57]. Furthermore, genomic aberrations, including deletions and rearrangements, are observed following repair of CRISPR-Cas9-induced damage [58]. In addition to these two sources of potentially oncogenic (and immunogenic) changes, the use of a lentiviral vector introduces the risks mentioned above (e.g., IM). As pointed out by the original authors [55], overexpression of *CD47* is also associated with malignant transformation; the inclusion of inducible kill-switches was proposed as a safety measure in this respect. Other vectors and gene-editing techniques [59,60] (or epigenetic/non-genetic controls on gene expression) could also be used to reduce the risks; however, the multiple genetic manipulations and additional expansions in culture require that UiPSC be subject to especially careful assessment. Similar concerns may surround genetically modified human iPSC-derived therapeutics, such as Fate Therapeutics Inc.’s FT500 (a line of derivate natural killer cells) which has recently been approved for clinical trial in the US as a treatment of advanced solid tumors [61].

## 3. iPSC Are Dangerous by Design?

### 3.1. Neoplasia Following Stem-Cell Therapies

The engineers of iPSC clearly intended them to be immortal cell-lines, and with such design comes a significant challenge to their safe translation to the clinic. Furthermore, two of the Yamanaka-factors, *c-Myc* and *Klf-4* are potent oncogenes [62]. In view of the nature of iPSC, reports of tumourigenesis in a mouse model following receipt of iPSC-derived neural cells [63], and in a primate model with undifferentiated iPSC [64], are not surprising. Teratoma formation with procine and bovine iPSC-derivates has been attributed to residual expression of reprogramming factors in the derivates [65]. More recently, evidence for transgene reactivation leading to proliferative growth in mesenchymal and endothelial iPSC derivatives generated from iPSC reprogrammed using integrative constructs in mouse models [66], has highlighted issues relating to iPSC-derivate stability.

There has been only one clinical test of iPSC in humans; however, adult Stem-Cell Therapies (SCT), involving either directed differentiation of adult multipotent stem cells (usually of fetal origin) or transplant of multipotent cells themselves, are commonly practiced worldwide. Re-differentiated adult stem cells are comparable to four features of iPSC that relate to their safety: (i) stem-cell character; (ii) being derived from clonal expansions of cells in culture; (iii) having been reprogrammed by certain factors; and (iv) being re-differentiated into a tissue type cell. Therefore, experiences with adult stem cells can shed some light on potential problems with iPSC. Receipt of mesenchymal, embryonic, and fetal neural stem cells, to regenerate damaged neural tissue, has been associated with development of apparently benign neoplasms resembling glioneuronal tumors. In one case a boy developed neoplasms in brain and spinal cord, detected four years following SCT (with human fetal neural stem cells) in Russia for ataxia telangiectasia [67], in another adult male, who was not taking immunosuppressants, developed a thoracic spinal cord neoplasm following SCT for ischemic stroke in China, Argentina, and Mexico [68]. Such SCT are subject to less regulation, if any [69], than those in the EU for example, and this may explain the lack of SAE in published hESC trials (see Table 2). Nevertheless, there is need for great caution as the reported neoplasms became apparent beyond the timescale of follow-up in hESC and iPSC clinical trials.

### 3.2. The Challenge of Removal of Undifferentiated iPSC

Neoplasia in animal models for iPSC-therapies has been attributed to a failure to remove all undifferentiated iPSC from the infusions, which most often leads to benign teratoma formation [73]. Indeed, there is evidence that incomplete differentiation is hazardous. For example, some cells sampled from two-week-old embryo-bodies formed from mouse ESC, exhibited a derivate phenotype (i.e., loss of pluripotency markers, hypomethylation of retrotransposons, and constituent of a differentiated monolayer), and yet recovered their ESC phenotype upon transfer to ES cell culture conditions [74]. Similarly, silencing of Yamanaka-factors such as *Sox2* is vital for retinal integration and suppression of neoplasia in mouse ESC-derived retinal progenitors [75]. Fortunately, various strategies have been developed to ensure the removal of incompletely differentiated and pluripotent cells from iPSC-derivate therapeutics.

The use of suicide-genes, such as *HSV-tk* (confers ganciclovir susceptibility), is a common approach to the removal of undifferentiated iPSC [76]. A better targeted alternative, which does not also eliminate the cells derived from iPSC, involves PluriSIns, Pluripotent-Specific Inhibitors, which attack unique features of the pluripotent metabolome. For example, PluriSIn SCD1 greatly inhibits HDF-derived iPSC, but not human telomerase reverse transcriptase immortalized HDF controls [77]. A cross-species alternative, to removal by antibodies or targeted toxins, is lectin-mediated removal which is robust to culture conditions, protocol and cell source [78].

Quantitative Reverse-Transcription Polymerase Chain Reaction (qRT-PCR)-based assays for undifferentiated hiPSC (as used in RIKEN’s FIH test) have a 1 in 10^5^ detection threshold [79] and, as 10^4^–10^5^ undifferentiated cells are required for tumourigenesis in immunocompromised rodents [80], these assays offer the required sensitivity, particularly if coupled with SNV screening [81]. Nevertheless, qRT-PCR can only detect residual hiPSC expressing a target marker (e.g., *Lin28* mRNA). Consequently additional epigenetic and differentiation-confirmation tests are required, for example methylated DNA immunoprecipitation-sequencing for genome wide epigenetic profiling [82], together with a panel of transcriptomic signals for re-differentiation markers characteristic of the intended cell type (e.g., ↑CD133, ↓*SSEA4* and *OCT4* for hiPSC-Neural Stem Cells (-NSCs) [83]).

Obtaining a pure product that comprises only differentiated cells may also be important for immunological reasons. T-cell responses to iPSC-derived implants received by syngeneic hosts were attributed to the presence of undifferentiated and/or partially differentiated cells [84]. In support of this, no response was found when in vivo differentiated iPSC (through iPSC-chimeric mice) were received by syngeneic mice, whereas ectopically transplanted in vitro differentiated iPSC-derived cardiomyocytes did evoke a T-cell mediated immune response [85]. Other work suggests that the T-cell response to undifferentiated iPSC is different from that mounted against their mature derivatives [86]. Furthermore, proteomic and epigenetic study suggests atypical innate immune response signaling in iPSC-derived cells [87]. In contrast, there are indications that autologous-derived cells are immunogenic, although this varies with tissue type (but not injection site); for example, hiPSC-derived-smooth muscle cells were highly immunogenic, whereas hiPSC-derived RPE cells were not [88]. It is clear that much more work is required to improve understanding of iPSC immunology to the level required to appreciate fully the clinical relevance of the responses observed in such studies.

### 3.3. Genetic Stability: iPSC Have a Good Safety Record

Follow-up of the FIH AMD test patient revealed no signs of adverse reaction [13]. A subsequent iteration of the RIKEN AMD iPSC-derived RPE implantation program, involving five patients receiving allogeneic iPSC from different donors, did report one SAE which required removal of the implanted sheet; however, this event is being viewed as caused by the surgical method used for cell transplantation rather than the iPSC implant [89]. Despite reports of genomic aberrations, none of the Pluripotent Stem Cell (PSC) clinical trials to date (Table 2) has reported a neoplasia-associated-SAE attributable to the transplanted cells. The first trial of an allogeneic iPSC, an iPSC-derived MSC infusion for the treatment of GvHD (Graft versus Host Disease) in Bone Marrow (BM) transplant patients, is now underway (Cynata Therapeutics: NCT02923375) and involves an estimated 16 participants [90]. A phase I trial (NCT01691261) involving two participants receiving an hESC-RPE monolayer, on a synthetic basement membrane, implanted into the sub-retinal space as a treatment for acute wet AMD, is nearing completion, and reported no SAE related to the hESC-RPE after 12 months follow-up. The trial is expected to be expanded to include a further eight patients [91]. Most recently, iPSC-derived dopaminergic precursor cells were implanted into the brain of a Parkinson’s Disease (PD) patient, with plans to treat six more patients by the end of 2020 [15]. In addition, further trials are planned, with a small-scale trial of iPSC-derived cardiomyocytes, for the treatment of ischemic heart disease, to begin in 2019 [92].

The absence of malignancy in iPSC-based interventions may in part be explained by the possibility that the genetic aberrations seen in iPSC are also common in non-PSC; for example, NSC, in independent laboratories, show T19, and MSC loss of chromosome 13 [93]. Mosaic aneuploidy in NSC, of both adults and developing neonates, is often observed [94,95], and is thought to function in the development and maintenance of neural diversity [96]. In addition, ~50% of CNV found in hiPSC are also found in source HDF [97]. Similarly, LINE-1 retrotransposons (a potential cause of IM), which are highly expressed in NSC and thought to increase diversity during neural development [98], may be activated during iPSC reprogramming [99]. Also, NSC with a 1q amplification did not form tumors when injected into immunocompromised rat brains [100]. Consequently, many of the aberrations seen in iPSC may in fact be beneficial and typical of normal stem cells [81].

The apparent lack of tumors attributed to hiPSC-derived cells in the face of notable genomic aberrations might also be consequence of their distribution. Studies of mouse and human iPSC have shown mutations to be concentrated in regions associated with structurally condensed lamina-associated heterochromatic domains, that is to regions unlikely to have a marked influence on the transcriptome [101]. A study of fibroblast-derived iPSC, reprogrammed by retrovirus, SeV, and mRNA-vectors, revealed Combined Annotation-Dependent Depletion [102] scores to be concentrated below 15 (i.e., the variants were likely to be non-deleterious) [33]. Similarly, study of 25 clinical-grade hESC lines revealed 15 large CNV, none of which was associated with a known clinical syndrome [3]. In addition to methylation status, variations in the physio-chemical properties of the chromosome may affect the reliability of mismatch and base-excision repair, with germline mutational cold spots characterized by purine tracts and hotspots showing alternating purine-pyrimidine bases (often featuring CpG islands) [103].

An additional factor limiting tumourigenesis may be that efficient reprogramming requires an intact HR pathway. Reprogramming’s marked effect on chromatin structure and rearrangement, and translation, suggests the necessary involvement of DNA repair pathways. Key HR genes, such as *Brca1*, *Brca2*, and *Rad51* appear to be required, even in protocols where *c-Myc* or viral-integration are not involved [104,105]. These observations suggest that highly proliferative (i.e., malignant) cells arising in culture may not reprogram successfully and so be less likely to make their way into any downstream therapeutic. Indeed, attempts to reprogram 13 different acute myeloid leukemia samples, with different genomic aberrations, indicated selection for normal genomes during reprogramming, as no hiPSC colonies possessing the donor aberrations were obtained [106]. In contrast, teratogenic hiPSC have been obtained from a blast crisis stage chronic myeloid leukemia-derived cell line, although other such lines tested resisted reprogramming [107]. There is also evidence that reprogramming requires functional Nucleotide Excision Repair (NER), as donor cells from Xeroderma Pigmentosum (XP) patients, yielded ultraviolet light (UV) hypersensitive XP-iPSC which showed an accumulation of SNV [108]. Estimation of mutation rates in somatic cells (~14 SNVs per cell per generation) and in iPSC suggested a ten-fold lower rate in the latter, which was attributed to persistence of HR activity of throughout the cell cycle in pluripotent cells [109]. In contrast other studies suggest that checkpoint activation and HR fail to initiate following replication stress in hESC, because these cells do not accumulate the required single-stranded DNA regions [110].

Finally, deep Next-Generation Sequencing (NGS) and similar high-fidelity sequencing is suggesting that SNV attributed to reprogramming and ex vivo expansion are pre-existing in the donor-cell population at very low frequencies as mosaic variants. NGS on clonal iPSC and their parent fibroblast population suggested that reprogramming is not mutagenic, as the iPSC-lines showed no more variants than did fibroblast sub-clones derived from the same parental pool [111]. Indeed, some regard all clonal SNV (i.e., those with allele frequency of approximately 50% and shared across lines) as originating in vivo and not associated with reprogramming [109,112,113]. NGS, coupled with Sanger re-sequencing, revealed 391 mutations that were shared by all iPSC lines derived from mono-clonal Endothelial Progenitor Cells (EPC) and the EPC themselves); however, 1644 variants unique to each iPSC-line, not present in the EPC at a detectable frequency, were also found and assumed to be induced by reprogramming and culture expansion [109].

The question of donor-inherited versus in vivo-acquired mutations is important as it relates not only to the safety of reprogramming, but also to the potential for clonal competition and cell-line evolution in culture (see Section 4.2). Several authors have used mutation type, in addition to allele frequency, to distinguish donor from de novo mutations. One study found that reprogramming-associated SNV showed a distinctive bias towards transversions [112], while other regarded UV-associated mutations (i.e., C > T and CC > TT mutations) as having occurred in the donor cells of skin-fibroblast-derived iPSC [113]. The accelerated cell growth and division following induction of the reprogramming factors is considered to effect a shift from oxidative respiration to oxidative glycolysis, which increases the potential for leakage of Reactive Oxygen Species (ROS) into the iPSC cytoplasm. ROS may oxidize nucleotides to produce species such as the mutagenic 7,8 dihydro-8-oxoguanine, capable of generating single- and double-strand DNA breaks [114], the signatures of which may also indicate non-donor variation. Indeed, it has been suggested that 8-oxoguanine associated mutations might be prevented by increasing the expression of key enzymes involved in the base-excision repair pathway, such as MTH1, OGG1, and MUTYH [114]. By contrast, deamination of methylated cytosine appears to be the main cause of mutations in vivo [109]. In addition, replication stress itself, seen as elevated numbers of stalled and collapsed replication forks, can lead to further genomic aberrations [115].

### 3.4. Clinical Trial History of MSC-Based Interventions Can Inform iPSC Safety Assessment

The lack of SAE observed in the iPSC trials so far could be a consequence of limited follow-up time. Indeed, the earliest iPSC human trial terminated a mere six years ago [73], and experiences with hESC-therapies have been included in this review to address this. In addition, some indications of potential iPSC safety may be gleaned from the safety record of MSC-based interventions. MSC, a catch-all term [116] that also includes (and might be predominantly) multipotent mesenchymal stromal cells, have been extensively used in regenerative medicine. MSC derived from iPSC have been investigated as a solution to the problem of the short lifespan of MSC during in vitro expansion [117]. In addition, CYP-001, the iPSC-derived MSC (see Section 3.3), has been found to be safe, and well tolerated in the first cohort (of eight GvHD patients) enrolled in phase I trials [118]. MSC are also informative in terms of the numbers of patients that have received such therapeutics without SAE. None of the 493 MSC trials have so far reported any SAE relevant to iPSC therapy, i.e., tumourigenesis [119]. A meta-analysis covering 36 studies, comprising 1012 participants, did not report any significant link between intravascular delivery of MSC and malignancy (or short-term SAE such as infection and organ system complications) [120]. Similarly, a systematic review of 844 procedures involving intra-articular injections of MSC found no association with such adverse events [121]. The lack of an association between MSC and malignancy is despite their implication in promoting stem-like properties in cancer cells [122]; this suggests that in the sense of their common features with iPSC, MSC are very safe. The comparative database for safety assessment of iPSC continues to expand, with 2835 patients currently enrolled in ongoing MSC clinical trials for cardiac diseases alone [123].

Like iPSC, MSC-therapies involve significant ex vivo population expansion because they tend to exhibit low frequencies in source tissues, for example comprising only 0.001–0.01% in the BM, and large infusions, of millions of cells per kg of patient body mass, are typically required [124]. Preparation of enough cells for a single therapeutic intervention can involve 3–5 passages, around 15 doublings, and up to one month in culture [125]. In this sense the potential for culture adaptation, genotoxic stress, accumulation of genetic aberration [126], and clonal heterogeneity and competition, is comparable with that of iPSC development. Unlike iPSC, however, MSC show loss of stemness over prolonged expansion, normally enter a senescence state characterized by cell-cycle arrest and loss of multilineage potential [127], and show therapeutic effects not dependent on persistence in vivo [124]. CD34+MSC are of particular interest, as CD34+ markers are here associated with enhanced and prolonged proliferative capacity [128], i.e., a more PSC-like phenotype. As CD34 is regarded as a marker of hematopoietic cells, the International Society for Cellular Therapy requires that ≤2% may express CD34 [129]. At such a level of stringency, the implantation of up to 4 × 10^7^ CD34 + MSC is possible for each adult treated with 2 × 10^9^ cells. In view of this, the MSC literature provides a wealth of advanced phase clinical trial data, involving thousands of patients and tens of billions of CD34+ cells administered over a period exceeding a decade, and captures two of the features of iPSC-derived implants (stem-cell characteristics, and expansion in culture). MSC-therapies do not inform the reprogramming and re-differentiation involved in iPSC-based product development; therefore, clinical deployment of iPSC should be gradual and accompanied by long-term, and detailed, follow-up.

## 4. iPSC Are Inherently Unstable and Unreliable?

### 4.1. iPSC May Possess Overt Cancer Driver Mutations As Well As Cryptic Tumourigenic Genetic Changes

Despite a large literature base supporting the view that single-nucleotide polymorphisms and CNV are not a threat [34,109,111,113,130,131], genomic aberrations are increasingly reported in hESC and iPSC-lines, and raise the possibility of de novo emergence of malignant neoplasia, particularly following receipt of autologous hiPSC-derived cells [132]. High-depth WGS of iPSC-lines revealed concentrations of SNV and CNV in stem-cell regulatory elements and binding sites of transcription factors, characterizing a regulatory landscape quite distinct from that of the founding cells [133]. Furthermore, WES of iPSC with ultra-deep amplicon sequencing of the parental fibroblasts, indicated that approximately 75% of mutations found in iPSC were acquired during cellular reprogramming and could be the result of oncogenic reprogramming factors and genotoxic stress [134]. This is in stark contrast to other WGS and WES studies of iPSC-lines concluding that non-germline SNV and short indels (insertions and deletions) arise mostly from the donor cells rather than during reprogramming [34,109,111,113,130,131].

Nevertheless, the observation of the same aberrations common to iPSC and ESC from different source tissues (i.e., they are unlikely to be the result of random drift) is particularly worrying, as this may indicate selection for more proliferative clones in culture. For example, the common sub-chromosomal duplication in chromosome-20q was found to include *Bcl-x L*, an anti-apoptotic gene whose overexpression is known to enhance the survival of hESC [135]. Duplication of chromosome-12 is also common [136]; this bears a *Nanog* pseudogene, which is the shortest region to be commonly duplicated in hPSC, suggesting that *NanogP1* is expressed and confers an advantage for hPSC in vitro [137]. Trisomy of chromosomes 1, 12, 17, and X (denoted T1, *et seq*), and amplification of 20q have been detected in up to 34% of hPSC-lines karyotyped [138]; such aberrations are also common in human carcinoma [139], which is a clear safety concern.

Failure to detect oncogenic mutations following iPSC reprogramming (see Section 3.3) is not necessarily a guarantor of the non-tumourigenicity of iPSC-therapies. Detection of oncogenic SNV depends on their prior recognition and inclusion in the cancer gene catalogues, which may not include all genes relevant to establishment of iPSC-derived cancers. Even if known a priori, it appears possible that oncogenic aberrations could evade detection. WES on 140 independent hES-lines, including 26 lines prepared following Good Manufacturing Practice (GMP), for potential clinical use, suggested that researchers have “unknowingly and routinely used hPS cells that harbored cancer-related missense mutations” [140]. The WES revealed that 5% of the lines were entirely heterozygous for a *TP53* mutation associated with cancer and autosomal dominant inactivation of P53, and five of the lines carried six mutations in *TP53.* In over 60% of cases the mutations were mosaic and so might not be detected by limited sampling for quality control. The *TP53* mutations exhibited allelic frequencies ranging from 7–40%, suggesting their presence in 14–80% of cells in culture [140].

Furthermore, studies of RiPSC-lines have shown a notable concentration of mutations at binding sites for Polycomb Repressive Complex 2’s catalytic component EZH2 [33], and increased EZH2 activity is known to effect H3K27me3 accumulation leading to a repressive chromatin state. Although it is not known if the SNV reported confer gain-of-function, activating mutations in the SET domain of EZH2 are known to be oncogenic [141]. *EZH2* up-regulation has been found in several cancers, including breast [142], bladder [143], prostate [144], and non-Hodgkin lymphoma [145]. EZH2 may also have suppressive roles as inactivating mutations are found in some cancers, e.g., T-cell acute lymphoblastic leukemia [146]. Interestingly, *EZH2* expression is correlated with *Myc* expression in prostate cancer [147]. Also of concern is the observation that SNV detected in study of iPSC were “generally benign” [81], as even a single oncogenic SNV, if conferring a proliferative advantage, may rise to very high frequency (even to fixation, i.e., 100%) in an iPSC cell culture.

Study of coding mutations in hiPSC-lines has indicated non-synonymous to synonymous substitution ratios in oncogenes similar to those in cancers, thus implying similar selection pressures on those genes in iPSC culture as in human cancers [34]. The same study revealed that all the SNV found were fixed in the iPSC populations; this may be a consequence of the population genetic bottleneck caused by hiPSC colony picking during cell-line production. The same bottlenecking could bring SNV occurring at very low frequency in the parent cell population to fixation in an iPSC-line [34]. The implication here is that hiPSC-lines may not represent the gene-pool of the patient’s own cells, and so be of limited use in understanding the disease process, or not representative of normal autologous tissues in implants. The problem is compounded by the fact that over 300 iPSC-lines would be required to account for inter-patient variation in study of a disease [133].

Several protocol changes have been proposed to avoid genetic aberration during reprogramming. As well as improving the programming efficiency of episomal-vectors, culture at physiologic oxygen levels has been shown to reduce the frequency of aberrations [148]. Another study has found a MefMech (feeder layer of mitotically inactivated MEF, passaged by mechanical disassociation of colonies) technique to allow long-term passage and expansion of hiPSC with no detectable aberrations [73], with similar improvement for hESC lines BG01 and BG02 [4]. A more innovative attempt at addressing genomic instability is the CryoPause approach. CryoPause allows synchronization of multiple hiPSC-lines, without need to maintain one while the others are expanded; they also avoid the need for multiple passages while safety assays are performed. CryoPause cells can be directly differentiated immediately after thawing, without need for recovery and expansion [149]. Further study is necessary to ensure that the CryoPause process itself does not induce aberrations, and that the method is reliable and robust.

Somatic Cell Nuclear Transfer (SCNT) may provide an alternative to iPSC that avoids the long periods in culture required by the reprogramming step, reduces the effect of epigenetic memory [150], and produces cells that transcriptomically and epigenetically better resemble the ESC of the fertilized embryo [151]. Human SCNT also shows high efficiency compared with hiPSC reprogramming (2–5% [152,153]); however, SCNT requires sourcing of oocytes and manipulation of preimplantation embryos. Furthermore, not all studies report greater genome stability in SCNT reprogrammed lines. A comparison of seven such lines with seven isogenic iPSC-lines found no significant difference in terms of mutation frequency or epigenetic markers [154]. Another study compared SeV-vectored iPSC from HDF with ESC produced by SCNT on the same somatic cell culture, and included IVF-ESC (sharing the same mitochondria as the SCNT-ESC); the iPSC and SCNT-ESC were found to resemble one another in transcriptome, epigenetics and cardiac differentiation efficiency [155]. The observation of similar levels of SNV between iPSC and SCNT-ESC, despite the latter’s shorter reprogramming time (generally 5–7 days [156]), suggests that most of the genetic aberration could be a result of reprogramming rather than of rapid cell division. As iPSC production is more convenient, further work is required to determine if SCNT-derived lines are in fact more stable.

### 4.2. The Problem of iPS-Cell Evolution during Preparation of Therapeutic Product

Reports of reduced efficacy of the 2016–2017 influenza vaccine against clade-3C.2a H_3_N_2_, following antigenicity altering mutations in chicken–egg-adapted viruses [157], serve to remind us of the potential for biotherapeutics to evolve during production. Although exhibiting a lower mutation rate, iPSC are at risk of similar “evolution in preparation”. Fully reprogrammed fibroblast-based hiPSC arise at a frequency ranging from 0.01 with SeV to almost 0.05 with mRNA [29], with iPSC forming in a sea of partially reprogrammed cells [158]; therefore iPSC-lines are based on tremendous clonal expansion. Indeed, there might be no need for isolation of iPSC by colony picking, as reprogrammed cells appear capable of outgrowing somatic cells within a few passages [159]. The FIH test of hiPSC required four weeks in culture to obtain hiPSC [11], followed by maintenance for >2 months to allow tumourigenicity testing―time in culture has been found to be a significant factor in the emergence of genetic abnormalities in hPSC [136,137]. Several studies have reported an increase in aberrations with time. Fluorescent in situ hybridization analysis of hESC revealed a rise in T17 between passage 22 and 39 from 76% to 95% [160], with similar reports for T12 [161]. The same is likely to be true for iPSC and, as detailed above, changes such as T12 can confer a proliferative advantage which would be selected for in culture. The precise culture conditions and source of cells may have some influence as, in a contrasting study [113], little indication of iPSC subclone evolution in culture over time was observed, with sub-clonal SNV showing constant frequencies throughout early and late passages, as well as during differentiation into cardiomyocytes. Other studies, in contrast, suggest that distinct mutational events do occur during reprogramming and are recognizable by a predominance of C>A transversions, which characterize ex vivo events [109].

The theoretical potential for iPSC evolution during production is quite strong, but depends fundamentally on estimation of the in vitro mutation rate. Rates, excluding donor-cell SNV, have been estimated for several human fibroblast iPSC-lines, with reported per bp per cell division (pbp/cd) values of 1.8 × 10^−10^ [109], 6.7 × 10^−10^ [34], 4.0 × 10^−9^ [134], and 1.6 × 10^−9^–7.8 × 10^−9^ [33], with rates from the latter two publications converted to pbp/cd. An average value of 2.4 × 10^−9^ is used here. The in vivo somatic mutation rate has been estimated as in the order of 0.3 × 10^−9^–1.0 × 10^−9^ [162]. Consequently, the mutation rate in iPSC production may be only slightly higher than that in vivo, but unlike most cells in the body iPSC are rapidly dividing. An average of 30 doublings is assumed, thus giving a final cell population of 10^9^ cells. The probability (*P*) of any randomly chosen cell escaping mutation is then approximately [163]:

*e*^−*n*N*r*^, (1)

where *n* is 30, N is 3 × 10^9^ (bps), and *r* is 2.4 × 10^−9^. The result from Equation (1) indicates that all cells in a typical iPSC-therapeutic are likely to have experienced at least one SNV. If 100,000 donor cells were reprogrammed as is common in protocols [159], rather than a single cell, the proportion of mutated cells in the final iPSC population would still be effectively 100%. The same conclusion was reached in earlier work using different starting parameter values [163]. To assess the risk of oncogenesis, the proportions estimated above must be corrected for the fact that most mutations will occur in genomic regions that are not associated with cancer. A search of the genome-mysql.cse.ucsc.edu, using MySQL (version 5.7.2.4 [164]), for genes listed in both tiers of the Catalogue Of Somatic Mutations In Cancer (COSMIC [165,166]), revealed a total transcriptome length of 82789992 bp (where a gene possessed multiple transcripts the longest one was recorded); this gives some indication of the minimum size of the oncogenetic target in the human genome (i.e., where an SNV needs to occur to have a chance of causing cancer), excluding non-transcribed regulatory regions. Adjusting values in Equation (1) for this smaller mutational target, and assuming 100,000 donor cells, still implies that over 90% of cells in a typical iPSC-line are expected to possess at least one mutation in a gene associated with the initiation of cancer. Finally, an additional correction is needed to accommodate the fact that cells die during culture; therefore, more doublings are required to reach the required 10^9^ cells. A per division death rate (*d*) would increase the number of required cell divisions according to Equation (2):

*n* = −log_2_(*N_f_/N_i_*)/*d* − 1, (2)

where *N_f_* is the final number of cells is the iPSC product, and *N_i_* is the starting number. The estimated rate of apoptosis for mouse-MEF-derived iPSC is reportedly 0.483 cells per day [167]. Such a death rate implies (through Equation (2)) around 58 days or 41 doublings (assuming a doubling time of 34 h [134]) to reach 10^9^ cells. Interestingly, this death rate is consistent with an estimate of 43 doublings as required to generate a human foreskin fibroblast-derived iPSC from a given reprogramming-target cell [134]. Assuming 41 doublings we expect that over 99.9% of cells will have experienced at least one mutation in a COSMIC listed (cancer) gene during reprogramming and colony expansion.

The above iPSC mutation rates clearly indicate the presence of the raw materials for evolution in preparation and culture adaptation. Reprogramming may also carry variants that were rare in the donor to high frequency in the hiPSC-line, thereby producing derivates that are not representative of a typical host cell upon re-transplantation. Indeed, a study of protein-coding mutations in hiPSC showed that near half of apparent mutations were in fact present in the donor-cell population at very low frequencies [34]. Nevertheless, it has been hypothesized that donor-inherited mutations are neutral because somatic cells are under high selection pressure, whereas those arising in cell culture and/or during reprogramming may include non-neutral SNV because there has been insufficient time for their fixation by positive selection or their removal by purifying selection [113]. Variants originating in the donor cells would be fixed, a population genetics term meaning that their frequency in the cell population is 1.0 (i.e., they are clonal mutations). Of course it is possible, as time in culture becomes long, that non-donor (de novo) mutations may become fixed. The observation that sub-clonal SNV had a significantly greater fraction of low-impact variants compared with clonal SNV [113] supports this possibility of fixation of de novo mutations in iPSC culture.

As mentioned in Section 4.1, an SNV conferring a proliferative advantage (either originating as a rare allele in mosaic donor tissue or as a de novo mutation arising early in iPSC reprogramming) could come to dominate an expanding iPSC culture through clonal competition. The average selective advantage of a driver mutation has been estimated at 0.4% for both glioblastoma multiforme and pancreatic adenocarcinoma [168]. The fate of a mutation under positive selection can be considered under a Wright-Fisher model [169,170] with a coalescent [171] structure [172]. If a colony expansion from 10^5^ cells to 10^9^ cells in 41 generations is assumed, then the corresponding exponential growth rate *r-d* is 0.219 per generation. Following Haldane [173], assuming constant population size and small selective advantage *s* (e.g., 0.004), the probability of ultimate fixation of the selected allele ≅2*s*. For an exponentially growing population the probability of fixation increases by 1 + *r*/*s.* Consequently, in the present case the probability that the SNV will become fixed in the iPSC-line would be approximately 45%. Studies in vitro suggest that inactivating *TP53* mutations may confer a selective advantage as great as 1.9-fold per passage [140]; this implies a 97% probability (*P*) of fixation for a highly selected allele (as, for large *s*, ln(1−*P*)/*P* = −(1 + *s*)(1 + *r*) [173]).

**Figure 4:**
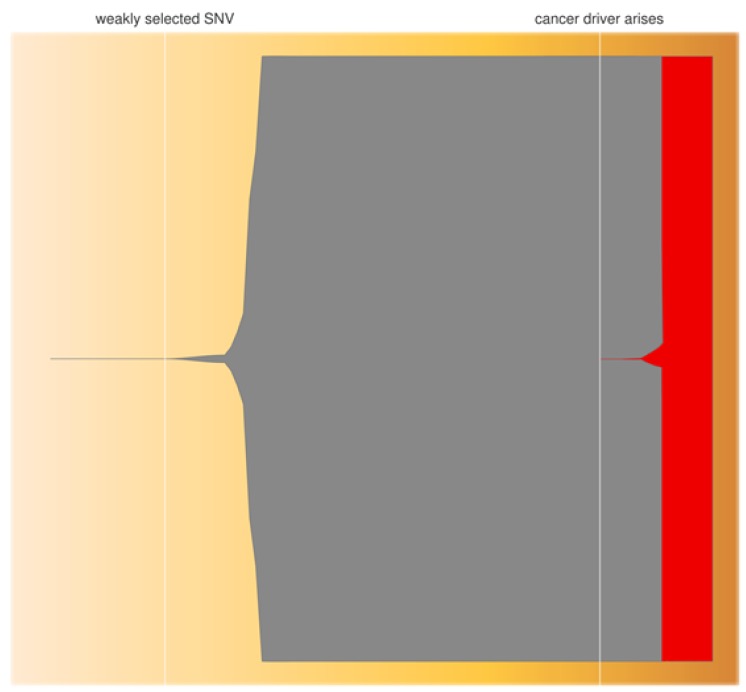
Clonal expansion of first a weakly selected Single-Nucleotide Variant (SNV) arising at generation two of iPSC in vitro culture (grey), followed by a second cancer driver mutation at generation 60 (red). The height of the expanding clones indicates allele frequency in the population. The weakly selected SNV reaches a frequency of 100%, replacing all cells bearing the donor-cell allele, but is then itself replaced by the more highly selected cancer driver which reaches 100% by generation 70. Plot produced using R package fish plot (version 0.5 [174]), with timings of mutations and rates of clonal expansion estimated using the parameters and equations in Section 4.2 (above).

The significance of combining the above predictions is that mutations in genes such as *TP53* will arise at some point during the production process with a probability over 99%, and when they do there is a similarly high probability of their coming to dominate the iPSC product (Figure 4)―a serious implication for iPSC clinical use. The above equations apply to SNV, indels, and other aberrations, including some forms of CNV, as long as they are stably inherited. Of course it cannot be assumed that all mutations at driver loci confer a growth advantage. Mutations may occur in inconsequential parts of the gene or be synonymous, and of course are more often deactivating than activating (i.e., non-oncogenic unless in a tumor suppressor gene). Nevertheless, such high probabilities of mutation and fixation in drivers, suggest that oncogenic mutations could arise at a low but significant frequency and, once they do, have the potential to greatly increase in frequency either before implantation or after―the former possibility increasing the chance of implantation of a malignant iPSC, thus leading to the latter. Equation (2) also assumes that linked subsequent mutations do not alter the selected haplotype to have a negative effect on growth, and that other driver mutations do not arise leading to clonal competition.

It is clear that long-term maintenance of hiPSC should be avoided in clinical applications. There is, therefore, a case for avoiding the use of SeV, as this requires around ten additional doublings to clear residual virus from the culture [29]. Similarly, protocols requiring relatively long periods for reprogramming, such as the electroporation-based methods (episomes and DNA minicircles) [175], maybe problematic. Aberrations may be reduced by restricting the use of older donor cells, or those particularly exposed to UV such as skin, and by culture at physiologic oxygen levels [41]. Some aberrations appear to be removed during culture, possibly through selection against damaged cells. The number of CNV in hiPSC was observed to decrease with increasing passage in culture [176]. Furthermore, proliferative ability tends to occur at the expense of differentiability―T8 mESC showed a selective advantage during in vitro culture, but rarely contributed to the germline upon chimaera formation [177]. Other studies have shown that CNV arising during reprogramming are lost at the stage of re-differentiation [178]. Consequently, rapidly dividing iPSC, although dominating the culture dish, may be removed by natural-selection or fail to re-differentiate and be removed by screening.

Selection may also occur at the re-differentiation stage, and is more difficult to detect karyotypically because the cells are often not dividing [81]. The first evidence of problems in re-differentiation came from large-scale SNV typing of WA07 hESC under directed differentiation into cardiomyocytes―by day five in culture, a marked predominance of T20 (or higher ploidy) cells was detected [137]. In another study, amplification of a region of chromosome-1q was shown to correspond to a loss of neuronal cell characteristics in hiPSC-derived NSC; however, when injected into immunocompromised rats these abnormal NSC did not form tumors, again supporting the view that many aberrations are not deleterious [100].

### 4.3. Epigenetics

A further problem related to differentiation is somatic memory. Epigenetic study of hiPSC has shown 71 Differentially Methylated Regions (DMR) relative to hESC [179]. Somatic memory is a particular safety concern as CpG methylation errors appear transmitted to hiPSC-derivates at high frequency, and could therefore affect the behavior of transplanted cells [180]. In addition, iPSC from different donor-cell types show distinct methylation patterns [181], and iPSC from blood cells more readily differentiate into hematopoietic cells and those from fibroblasts towards the osteogenic pathway [182]. Unlike karyotypic aberrations, however, DMR appear to decrease with passage number; though at reprogramming hiPSC appear to enter an initial stage of hypermethylation followed by waves of aberrant methylation that gradual decrease in amplitude until an asymptotic approach to hESC methylation profiles. By passage 40 there remained 100 DMR, with little improvement thereafter [181]. Lyonization may also diminish during prolonged culture, with X-inactive specific transcript expression and foci of H3K27me3, as well as expression of genes on the inactivated X-chromosome [183]. Nevertheless, lyonization in hiPSC appears more complete than in hESC [184], and of 87 imprinted hiPSC loci studied, only *Meg3* (6/15 lines) and H19 (all lines) showed aberrant methylation. Others regard cell function in hiPSC-derived stem cells as most likely robust to minor aberrations in methylation [185], and if so hiPSC are likely to perform normally in vivo. More work is required to address questions related to somatic memory before the behavior of iPSC-therapeutics can be fully understood.

The expansion of clones bearing driver mutations during iPSC production may be used as a means of detecting common problematic mutations. Monitoring of iPSC during development will allow selection coefficients to be estimated for commonly occurring SNV, such that the process of culture adaptation can be better understood. As the frequency of oncogenic SNV is likely to be lower than the theoretical predictions above (most mutations will be neutral or inhibit proliferation rather than drive it), it is likely that all common oncogenic SNV can be identified and iPSC-lines can be regularly screened for their presence. The results of studies into recurrent mutations, across independent iPSC-lines, are beginning to accumulate [101,112,113,186,187]. The increasing frequencies in T12 and T17 observed with hESC are consistent with ongoing positive selection in vitro. In further support of selection for rapidly dividing hiPSC is the observation that hotspots of aberration in the hiPSC genome are syntenic with hotspots in PSC of other species (e.g., human 17q25 and murine 11qE2; and macaque 16q and human 17q) [188]. The recognition of such common aberrations may lead to the development of a database of mutations to be targeted by Quality Control (QC) screening of iPSC and thereby enhance their safe translation.

Relatively little work has been done on the (epi-)genetic effects of re-differentiation, and the safety issues are not understood. The safe translation of hiPSC will require further studies in this area given the regulatory climate in which hiPSC are likely to be deployed. For example, the use of Food and Drug Administration-approved hiPSC will probably simplify the regulatory approval process, but such lines are likely to be older and culture-adapted.

## 5. Translation into Standard of Care Will Be Problematic?

Quality assurance requires standardized manufacturing processes delivering robust and consistent high-quality hiPSC. Unfortunately, even a minor change in the expression cassette ordering of reprogramming factors between laboratories appeared to affect the imprinting of the *Dl1-Dios3* cluster and the ability, or lack thereof, to form viable all-iPSC mice [189]. Clearly international harmonization is essential in ensuring safety, as are bespoke databases for traceability, and availability of lines of cGMP-compliant differentiated cells (for evaluation) [190]. Master Cell Banks (MCBs) such as FT500 [61] (see Section 2) can provide cost-effective mass-production of genetically modified cells and an off-the-shelf product that is readily available. Nevertheless, MCBs may not offer the expected consistency and uniformity. Stochastic events occurring during reprogramming, colony expansion, hiPSC selection, re-differentiation, hiPSC-derivate expansion and purification, storage, and transport are likely to complicate efforts towards a standardized product. Consequently, variation may exist within any MCB established for validation purposes, and between MCB and hiPSC product in the clinic. Such variability requires continual genotypic, phenotypic, and functional assessment. For example, after two weeks’ passage, re-differentiated MEF-originated iPSC-Embryoid-Body-derived-cardiomyocytes showed a similar proliferation rate to the source iPSC, and at four weeks, both derived cardiomyocytes and source iPSC formed teratomas; this suggests significant de-differentiation of the cardiomyocytes [191].

Although the problems appear insurmountable, several inroads have already been made towards taming iPSC for clinical use. Partial Reprogramming (PR) is a distinct solution to the problem of teratogenicity. PR preserves the proliferative and regenerative capacities of adult stem cells in vitro culture, resulting in cells at a midpoint between multipotency and pluripotency [192]. For example, peripheral blood monocytes have been converted to immature β-endocrine cells by growth-factor-induced PR [193]. Finally, the move away from xeno-supportive feeder cells (e.g., MEF) towards xeno-free and feeder-free surfaces is increasing the capacity for standardization and reducing immunogenicity and risk of pathogen transfer. For example, nanocrystalline graphene provides a fully synthetic surface that equals MEF in supporting culture of hiPSC [194]. More work is warranted to bring PR cells to the same stage as iPSC-derived cells, to provide a potentially safer, quicker, and cheaper source of material for cell replacement strategies.

Reprogramming-associated point mutations are regarded by some as a general feature of hiPSC [195]. As detailed in Section 4.2, a database of common genetic aberrations associated with hiPSC development can be compiled and used in QC screening at reprogramming, colony expansion and implantation stages, and after any process that forces the hiPSC population through a bottleneck (e.g., gene editing). Deep sequencing of donor cells is also required to detect pre-existing low frequency mosaic variants that may expand during iPSC production. It has been suggested that a threshold of 10^−11^ or 10^−12^ mutations per base pair per cell division should be set, beyond which an hiPSC should not be allowed in clinical use [163]. In addition to simply reducing the number of generations in cell culture by adopting strategies such as CryoPause, and using younger or potentially more genetically protected donor cells than fibroblasts [195], measures are being found to stabilize better the genome during reprogramming. For example, reprogramming in the presence of oocyte factor Zscan4, in combination with OKSM, was reportedly associated with not only improved genetic stability but also greater efficiency [196]. A similar effect was seen after increasing the level of checkpoint kinase 1 (CHK1) [197]. A further development is the availability of alternative factors to *c-Myc* [198]. Together such advances are greatly improving genome stability during reprogramming and paving the way to reliable and routine clinical deployment.

## 6. Conclusions

Clinical deployment of hiPSC offers a solution to the ethical and availability issues surrounding the use of hESC, and potentially also to the immunological problems associated with allogeneic stem-cell transplantation. The present review has highlighted experiences with currently practiced therapies involving endogenous stem cells, studies identifying genetic aberrations found in iPSC, and theoretical predictions based on population genetic theory―all suggesting a potential for oncogenesis associated with iPSC implantation that might be a barrier to widespread adoption of hiPSC-based therapies. In contrast, widely adopted SCT and the follow-up of the small number of hiPSC implantations made to date in humans, indicate that such therapies are safe. The reasons for this have been discussed, and include exclusion of cells with oncogenic potential (e.g., those with impaired DNA damage repair pathways) effected by the re-differentiation process, that epigenetic aberrations found in some studies may diminish with culture time, leaving the final product with a normal (hESC-like) methylome, that the genomic aberrations found in hiPSC are beneficial and typify the normal development of stem cells in vivo, and that mutations seen are synonymous, neutral or growth-inhibitory, or concentrated in structurally condensed (i.e., inactive) regions of the genome. Taken together, these findings suggest that iPSC-based therapies are likely to perform well in the clinic, particularly after refinement of production protocols in the light of future pre-clinical studies.

Protocol refinement is likely to be through a better understanding of the reprogramming process, together with a greatly expanded knowledge base (amassed through study of animal models and clinical experience); this will also lead to improved tests for undifferentiated cells. Processes such as the natural down-regulation of reprogramming factors in host cells, DNA repair mechanisms, and replicative-stress reduction will be exploited to refine hiPSC-derivate production. Technical innovations such as CryoPause will avoid lengthy culture steps during which iPSC may “evolve”, and synthetic (xeno- and feeder-free) culture systems are already improving standardization. As mentioned above, hiPSC selected for rapid growth in culture often fail to re-differentiate and are removed during purification of the hiPSC product. Carefully conducted hiPSC experiments have not shown tumourigenesis, and with continual improvements in detection of undifferentiated cells, the safety of hiPSC is likely to reach standards of GCP.

Standard of Care (SoC) must consider the long-term effects of genetic instability and gene alterations caused by reprogramming―even if detected aberrations are unrelated to disease-loci, protein production, or directly causative of cancer―to exclude or greatly minimize the risks associated with their use. Issues such as the relationship between alterations in the sequences (e.g., de novo mutations) at certain regions of the genome and inflammation or immune response has not been considered due to the relatively short follow-up time post transplantation and the low number of clinical studies to date. How mutations, epigenetic changes or alterations in non-coding regions are related to inflammation remains poorly understood [87]. Nevertheless, potential tests for immunogenicity of autologous hiPSC-derivates are already beginning to emerge in the form of screening for markers associated with aberrant immune signaling (e.g., overexpression of TLR3 short-isoform [87]) or directly for immune response (e.g., use of humanized mouse models [88]).

Consequently, the future is likely to bring many more clinical trials involving hiPSC in conditions such as AMD, PD, acute spinal cord injury, stroke, and acute myocardial infarction that lead to SoC interventions at least as safe as currently licensed advanced therapy medicinal products. Such trials will be supported by improved understanding of hiPSC epigenetics and improved tests for cells bearing oncogenic changes. Such hiPSC-derivate therapeutics will likely be directed against the increasing barrage of degenerative conditions facing an aging population.

## Figures and Tables

**Table 1:** Reprogramming strategies developed to avoid transgene integration, and most commonly used in the production of iPSC. Abbreviations: cGMP, Good Manufacturing Practice certified; EBNA, Epstein–Barr Nuclear Antigen 1; miR, micro-RNA. Sources used in compilation of the table are given in the rightmost column.

Procedure	Caveats	Source
mir-200c, 302s and 369s (direct)	efficiency 0.01% cf. 0.02% adenovirus and 0.27% retroviral	[26]
mRNAs (direct transfection)	1.4–4.4% efficiency, but high in vitro cytotoxicity, fails with hematopoietic cells	[27]
non-integrating adenoviruses	transfected hepatocytes (show high permissivity to adenovirus)	[28]
OriP/EBNA episomal plasmids	0.006–0.1% (with EBNA mRNA coexpression and hypoxia) cGMP	[29]
Sendai-viral (SeV)	efficiency 0.077%, but complex protocols	[30]
Small molecules (e.g., epigenetic regulators)	usually require one transgene (e.g., VPA, CHIR99021 and 616452 + *Oct4*), non-persistent	[31]

**Table 2:** Completed clinical trials involving pluripotent stem cells, with number of participants treated (N) and termination date. Trials listed are phase 1 or 1/2. Abbreviations: AL, Allogeneic; AMD, Age-Related Macular Degeneration; ASCI, Acute Spinal Cord Injury; AU, Autogeneic; CD15+ Isl-1+ CardioVascular Progenitors, CVP; First-In-Human, FIH; Ischemic Heart Disease, IHD; Oligodendrocyte Progenitor Cells, OPCs; Retinal Pigment Epithelium, RPE; Serious Adverse Event, SAE; Stargardt’s Macular Dystrophy, SMD.

Date	Agent (N)	Condition	Derivate	Comments
2011 AU	hESC (4)	ASCI	OPCs	Geron: effect remyelination; no SAE; early termination on financial grounds or futility; not reproducible; contains xeno-derived components (e.g., Matrigel) of potential immunogenicity [70]
2013 AU	Hesc (?)	ASCI	OPCs	NCT01217008 (Asterias Biotherapeutics): continuation of Geron’s phase 1 trial; completed but unpublished
2017 AU	hiPSC (FIH)	AMD	RPE	RIKEN: RPE engraftment to effect photoreceptor rescue; no SAE at 27 months; degeneration only halted; costly $930,000 [11]
2015 AL	hESC (9)	AMD	RPE	NCT01344993: RPE engraftment to effect photoreceptor rescue; no SAE at 12–37 months; visual acuity gain in 6 eyes at 6 months [71]
2015 AL	hESC (9)	SMD	RPE	NCT01345006: RPE engraftment to effect photoreceptor rescue; no SAE at 12–37 months; visual acuity gain in 3 eyes at 6 months [71]
2018 AL	hESC(6)	IHD	CVP	NCT02057900: Epicardial delivery of hESC-derivates to improve systolic motion in severe ischemic left ventricular dysfunction; no SAE at 18 months [72]
